# Space and habitat use by wild Bactrian camels in the Transaltai Gobi of southern Mongolia^☆^

**DOI:** 10.1016/j.biocon.2013.11.033

**Published:** 2014-01

**Authors:** Petra Kaczensky, Yadamsuren Adiya, Henrik von Wehrden, Batmunkh Mijiddorj, Chris Walzer, Denise Güthlin, Dulamtseren Enkhbileg, Richard P. Reading

**Affiliations:** a Research Institute of Wildlife Ecology, University of Veterinary Medicine, Savoyenstrasse 1, A-1160 Vienna, Austria; b Institute of Biology, Mongolian Academy of Science & Wild Camel Protection Foundation in Mongolia, Jukov Avenue 77, Bayanzurkh District, Ulaanbaatar 21035, Mongolia; c Leuphana University Lüneburg, Centre for Methods, Institute of Ecology, Faculty of Sustainability, Scharnhorststr. 1, C04.003a, 21335 Lüneburg, Germany; d Great Gobi A Strictly Protected Area Administration, Bayantoorai, Mongolia; e Departement of Wildlife Ecology and Management, University of Freiburg, Tennenbacher Strasse 4, 79106 Freiburg, Germany; f Denver Zoological Foundation, 2300 Steele St., Denver, CO 80205, USA

**Keywords:** *Camela ferus*, Mongolia, Satellite telemetry, Movement patterns, Habitat use, Wild Bactrian camels

## Abstract

Wild Bactrian camels (*Camela ferus*) are listed as Critically Endangered by the International Union for Conservation of Nature (IUCN) and only persist in some of the most remote locations in northern China and southern Mongolia. Although the species has been recognized as an umbrella species for the fragile central Asian desert ecosystem and has been high on the conservation agenda, little is known about the species’ habitat requirements, with most information coming from anecdotal sightings and descriptive studies. We compiled the only available telemetry data from wild camels worldwide. Seven wild camels, which were followed for 11–378 monitoring days, covered a total range of 28,410 km^2^, with individual annual ranges being >12,000 km^2^ for three animals followed over a year. Camels reacted strongly to capture events, moving up to 64 km from the capture site within a day, whereas normal average daily straight line distances were 3.0–6.4 km/day. Camels showed a preference for intermediate productivity values (NDVI, habitat type) and landscape parameters (distance to water, elevation) and an avoidance of steep slopes. Our telemetry results suggest that wild camels still range throughout the entire Great Gobi A Strictly Protected Area (SPA), are highly mobile, and very sensitive to human disturbance. Their habitat preference may be a trade-off between dietary and safety requirements. Small sample size did not allow the development of a full habitat model testing all variables simultaneously and we urgently call for more data from additional wild camels as a foundation for evidence driven conservation actions.

## Highlights

• Wild Bactrian camels persist in some of the most remote desert locations in northern China and southern Mongolia.
• Individual annual home ranges in the Mongolian Gobi were > 12,000 km^2^ and average straight line distances 3.0-6.4 km/day.
• Wild camels preferred intermediate productivity values and landscape parameters, but an avoided steep slopes.
• Wild camels still roam the entire Great Gobi A Protected Area, are highly mobile, and very sensitive to human disturbance.
• More data from additional wild camels as a foundation for evidence driven conservation actions is urgently needed.

## 1 Introduction

Wild Bactrian camels (*Camela ferus*) are listed as Critically Endangered by the International Union for Conservation of Nature (IUCN) and only persist in three locations in northern China (one in the Taklamakan- and two in the Lop Nur Desert) and one location in southern Mongolia (Transaltai Gobi; Hare, 2008). The species’ distribution in Mongolia is reported to have shrunken by ∼70% since the last century, and possibly as early as the 1940s, and became restricted to the area of today’s Great Gobi A Strictly Protected Area (SPA) in the Transaltai Gobi by the 1970s (Adiya et al., 2012; Bannikov, 1975; Zevegmid and Dawaa, 1973).

Wild camels roam some of the most remote corners of the central Asian deserts and despite early interest in their conservation (Hare, 1997, 1998; McCarthy, 2000; Reading et al., 1999; Tsevegmid and Dashdorj, 1974; Tulgat and Schaller, 1992; Zevegmid and Dawaa, 1973; Zhirnov and Ilyinsky, 1986) little is known about the species. Most information has been coming from anecdotal sightings and short-term or observational studies (Adiya et al., 2006; Dovchindorj et al., 2006a,b; Tulgat et al., 2002; Zhirnov et al., 2011). Several factors have inhibited attempts to gather more rigorous data on wild camels, including their extremely shy and elusive behavior (McCarthy, 2000; Tulgat and Schaller, 1992; Zhirnov and Ilyinsky, 1986), the remoteness, harshness, and vast expanses of the environment they inhabit, and the lack of access to or ineffectiveness of research approaches typically used elsewhere (e.g., light aircraft, and satellite telemetry). Even population estimates remain disputed, but with general consensus that wild camel populations are declining or are at best stable, primarily because recruitment appears low (Adiya et al., 2006, 2012; Hare, 2008; McCarthy, 2000; Reading et al., 1999).

Several factors are assumed to threaten wild camel persistence, including human disturbance, poaching, and competition from, hybridization with, and disease transmission from domestic camels (*Camelus bactrianus*) (Blumer et al., 2002; Mijiddorj, 2002a; Silbermayr and Burger, 2012; Tulgat, 2002). Increasing human encroachment into remaining camel range includes increasing numbers of herder camps and livestock density in the buffer zone of the Great Gobi A SPA (Enkhbileg et al., 2006), and escalating incidents of illegal placer mining (“ninja mining”) within the protected area (Adiya, 2008a). Although the Mongolian government prohibited the hunting of wild camels in 1930, some limited poaching still occurs (Mijiddorj, 2002a). Other threats to wild camel conservation suggested by various conservationists include habitat fragmentation by the Mongolian–Chinese border fence, climate change resulting in drying oases and deteriorating water and forage quality, food shortages during increasingly frequent “dzud” winters (various situations of harsh winter conditions), and wolf predation on young camels (Clark et al., 2006).

In Mongolia the species is recognized as an umbrella species for Mongolia’s desert ecosystems and is of high conservation interest, which resulted, among other things, in the creation of the 44,000 km^2^ Great Gobi A SPA in 1975. More recent conservation measures have focused on reducing the potential for hybridization with domestic camels through legislation changes enabling the removal of domestic camels from the protected area, discouraging the possession of hybrid camels, and marking and tracking of known hybrids (Enkhbileg et al., 2006). Additionally, regular ranger patrols, oasis restoration (Oyunsuren and Munkhgerel, 2006), occasional supplementary feeding with hay during harsh winters, establishment of a semi-captive breeding herd of wild camels near the Great Gobi A SPA headquarters in Bayantoori (Enkhbileg et al., 2006; Mijiddorj, 2002b), and wolf control (McCarthy, 2000) have been suggested and partially implemented. However, without measures to monitor camel population dynamics or track individual camels, the efficacy of these measures on the wild camel population remains largely unknown. For evidence-based conservation actions (Sutherland et al., 2004), understanding what factors influence camel movements or constitute critical camel habitat is crucial.

In 2001 and 2002, we equipped the first two wild camels with satellite collars to collect data on movement patterns and habitat use. Those animals proved very difficult to capture and technical problems compromised data collection. Nevertheless, those data provided our first objective insight into the large spatial requirements of individual camels (Reading et al., 2005). Further collaring attempts occurred in 2005 and 2007 overcoming the difficulty of capturing wild camels (Walzer et al., 2012), however technical problems in data acquisition prevailed (Kaczensky et al., 2010). In this manuscript, we compiled the only available telemetry data for wild camels worldwide and analyzed it against a detailed, large scale digital habitat database. We discuss the results in the context of the most recently debated conservation needs for wild camels in Mongolia.

## 2 Study area

Great Gobi A SPA covers 44,000 km^2^ in the Transaltai Gobi of southwestern Mongolia and was established in 1975 to protect the unique desert environment that provides habitat to several rare or globally threatened wildlife. A special focus had been on large mammals, particularly wild Bactrian camel, Gobi bear (*Ursus arctos gobiensis*), snow leopard (*Uncia uncia*), argali wild sheep (*Ovis ammon*), and Asiatic wild ass (*Equus hemionus*), all of which are listed in the Mongolian Red List of Mammals (Clark et al., 2006; Reading et al., 1999; Zhirnov and Ilyinsky, 1986). In 2004 the wild camel population in the Great Gobi A SPA was estimated at 350 individuals (Hare, 2008); although few data underlie this number.

**Fig. 1:**
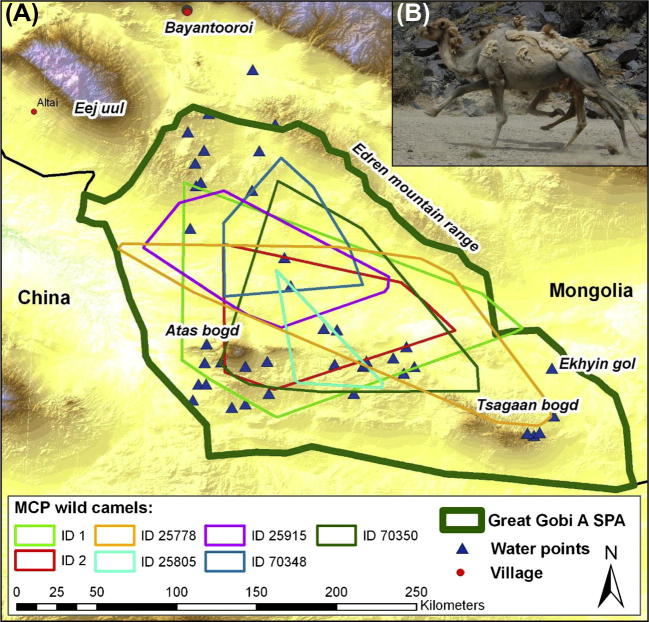
(A) Home ranges, expressed as 100% minimum convex polygons (MCPs), of seven wild camels monitored 2002–2007 in the Great Gobi A SPA in southern Mongolia. (B) Two wild camels running from disturbance by research jeep.

Elevations range from 525 m to 2683 m and the protected area encompasses large, mostly unvegetated depressions, extensive hill country, and several mountain ranges. The highest mountains are Atas Bodg (2695 m) in the southwest and Tsaagan Bogd (2480 m) in the southeast. Eej Uul Mountain and the Edren Mountain Range flank the northeast and China borders to the south and west of the Great Gobi A SPA (Fig. 1).

Great Gobi A SPA experiences a strongly continental climate with four distinct seasons: spring (March–May), summer (June–August), autumn (September–November), and winter (December–February). The average annual temperature is around 5 °C, but daily means may reach 40 °C in summer and drop to −35 °C in winter. Large parts of the protected area receive less than 50 mm of annual precipitation (interpolation from Hijmans et al., 2005, http://www.diva-gis.org/climate). Precipitation falls mainly during summer, but varies greatly between years and regionally, resulting in considerable fluctuations in vegetation cover.

Vegetation is scarce and in large parts dominated by drought-adapted central Asian desert elements, particularly woody Chenopodiaceae like saxaul (*Haloxylon ammodendron*), *Iljina regelii*, and *Anabasis brevifolia*. Open water is restricted to about 40 springs (not all of which are permanent), primarily located in or near mountain ranges. Lush oasis vegetation surrounds several springs and consists of reed beds (*Phragmites australis*), poplar trees (*Populus euphratica*), and tamarisk (*Tamarix ramosissima*) stands (von Wehrden et al., 2006a, 2009). Pasture productivity is primarely precipitation driven and subject to high intra- and interannual fluctuations (von Wehrden et al., 2012).

The park administration is located in Bayantooroi, about 50 km to the north of the park boundary. Human and livestock presence in the park is minimal, only 3 military posts in the south, ∼40 winter camps along the fringes of the Edren range, and ∼10 families at Ekhyn gol, graze livestock (sheep and goats, horses, cattle, and domestic camels) also in the protected area. However, the number of herder families in the buffer zone has increased dramatically during the past 30 years, and in 2004 some 444 families with 218,543 livestock had already registered to use this area (Enkhbileg et al., 2006). Due to the nomadic nature of livestock herding in Mongolia, herder camps occupation is highly variable in time and space within and among years.

The past 5 years have also seen a marked increase in illegal placer mining activities (open pit mining of alluvial gold deposits; Grayson, 2007) in and around the protected area.

## 3 Methods

### 3.1 Capture and telemetry

Between October 2002 and June 2007, we captured and radio collared 12 wild camels by free-range darting from a jeep (for details see Blumer et al., 2002; Reading et al., 2005; Walzer et al., 2012). All camels were captured out of herds of 3–10+ adult camels and only one camel was collared per group. Wild camels seem to live in open fission fusion groups, which tend to concentrate during the rutting season in winter (Adiya et al., 2006; McCarthy, 2000). However, data on group membership or stability of camel groups is lacking and thus we have no information which, and how many other camels each collared animal represents.

Due to three complete collar failures (see Kaczensky et al., 2010), one animal in poor physical condition (Reading et al., 2005), and one mortality (Walzer et al., 2012), we only collected location data for seven individuals; four males and three females (Table 1, Appendix A). We equipped the first wild camel with a Doppler-based Argos collar (Reading et al., 2005), but all subsequent animals received global positioning system (GPS) collars that used the Argos satellite system only for data transfer (GPS-Argos collars; Kaczensky et al., 2010). Of the seven collared camels, only three operated over an entire year or close to a year and regularly collected the quantity of data we anticipated (Appendix A; for descriptions of technical problems see Kaczensky et al., 2010; Reading et al., 2005).

### 3.2 Vegetation mapping

Nineteen plant (sub)communities for the Great Gobi A SPA have been identified and described using supervised classification of Landsat imagery (von Wehrden et al., 2006a,b, 2009). We reclassified these plant communities into seven main habitat types: (1) *Oases vegetation*, (2) *Higher and intermediate dry steppe*/*shrub communities*, (3) *Desert shrub communities*, (4) *Haloxylon semi-deserts*, (5) *Salty Haloxylon semi-deserts*, (6) *Iljinia deserts*, and (7) *Nitraria salt shrub stands*. Average productivity of the main habitat classes decreases from 1 to 7 (von Wehrden et al., 2006a). Single habitat types cover varied from a minimum of 1.1% for *Oasis vegetation* to a maximum of 59.6% for *Higher and intermediate dry steppe*/*shrub communities* being (Appendix B).

### 3.3 Other habitat variables

We downloaded Shuttle Radar Topography Mission (SRTM) tiles of 90 m resolution for Mongolia and northern China (http://glcf.umiacs.umd.edu/) to extract information on elevation and slope. We obtained 16-days Normalized Difference Vegetation Index (NDVI) layers of 250 m resolution from the Warehouse Inventory Search Tool (WIST) data center (https://wist.echo.nasa.gov/api/) as a proxy for pasture productivity (Kawamura et al., 2005; Kogan et al. 2004). We received GPS locations for all sources of permanent water from the Great Gobi A SPA administration to calculate distances to camel locations.

For all visualizations and spatial analysis we used ArcMap 9.3 (ESRI, Environmental Systems Research Institute, Inc., Redlands, California, USA) and the Hawth’s Analysis Tools extension (http://www.spatialecology.com/htools/).

### 3.4 Data analysis

#### 3.4.1 Space use and movement patterns

We used the term “home range” to indicate the total area covered during the entire observation period, and calculated this area as 100% minimum convex polygons (MCPs). We plotted MCP size for each wild camel against date to visually check whether camel home ranges reached an asymptote during our monitoring period, as a rough predictor whether camel ranges can be expected to further increase with longer monitoring periods. We also calculated the total area covered by all camels as the 100% MCP of all camel locations and visualized potential seasonal shifts in range use by plotting camel locations (pooled by year) on separate maps for spring, summer, autumn, and winter. We further explored potential seasonal shifts by calculating the mean net displacement of daily locations from a common reference point at the northernmost corner of the SPA.

We calculated the average distance travelled within 24 h (daily distances) for the four camels with regularly spaced GPS fixes (at 7 or 11 h intervals, Table 1) by calculating the straight line distance between those fixes that were 21–22 h apart and subsequently multiplied them with 24 divided by the actual interval assuming a linear relationship. We tested for individual differences using an ANOVA.

#### 3.4.2 Habitat use analysis

We defined habitat available to camels at different scales by drawing buffers of 5–25 km radii around each camel location for availability and randomly generated three pseudo-absence points within each of the five buffers. We choose the 5–25 km scaling since larger buffers led to non-significant effects and/or significant autocorrelations within the models. Given the large intra- and interannual changes in pasture productivity, we implemented a time specific approach by assigning animal locations to the relevant 16-days NDVI product. We extracted habitat and time-matched NDVI values for each animal location and its corresponding random points at the five different scales.

The high mobility of wild camels suggested that they could reach most regions within the Great Gobi A SPA within 24 h. However, camel locations were collected at variable intervals, often resulting in 2 or 3 locations per day (Table 1). To minimize temporal pseudo-replication within the dataset of individual camels while retaining all available information, we reduced the weight of successive GPS locations separated by less than 24 h by their time in hours since the last location divided by 24 h. All locations spaced ⩾24 h were given a weight of 1.0. Subsequent model inspection did not reveal any more temporal pseudo-replication effects nor did Moran’s I correlograms of model residuals suggest significant spatial autocorrelation.

We processed all predictors into ASCII files using ArcMap 9.3 (ESRI, Environmental Systems Research Institute, Inc., Redlands, California, USA) and imported them into the statistics program R (R Development Core Team, 2011). Extracted values for modelling where centered (with function scale) and scaled (to a mean of zero and a standard deviation of one) to make model estimates more comparable.

We used binomial generalized linear mixed models (glmm) with Restricted Maximum Likelihood (REML) optimized estimates. However, we were unable to construct a full model as the low number of sampling units (individual camels) and the spatial and temporal heterogeneity of the locations prevented full models to converge. To gain some insight into the importance of the different predictors we finally tested each predictor individually for each buffer size using the individual animal as random factor. To minimize potential overdispersion we implemented a random factor that contained as many different factors as total observations and nested it into the animal factor (Bolker, 2010). For the models using NDVI as predictor, we additionally included the 16-days NDVI interval as random intercept.

We tested all numeric predictors for linear and a quadratic (unimodal) relationships (also retaining the linear relationship). We tested quadratic relationships because other studies had shown ungulates to select for intermediate values due to various trade-offs (e.g., Creel et al., 2005; Mueller et al., 2008; Singh et al., 2010). For the categorical variable, we tested preference using the most common higher and intermediate dry steppe/shrub communities as reference category.

## 4 Results

### 4.1 Space use

#### 4.1.1 Total and seasonal MCPs

**Fig. 2:**
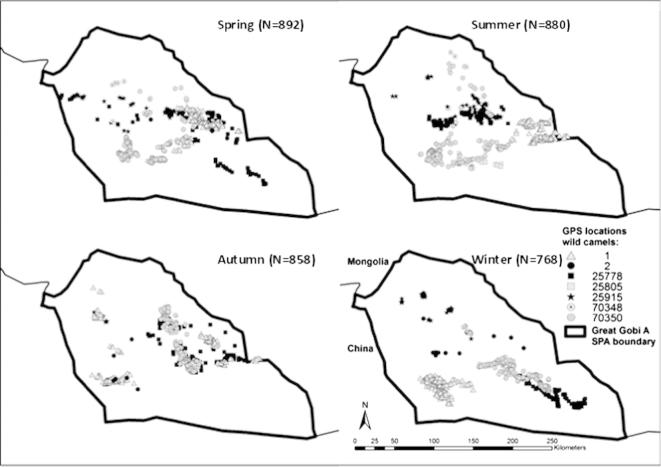
Seasonal pattern of wild camel locations in the Great Gobi A SPA from 2002 to 2007.

The seven wild camels occupied non-exclusive ranges of 1979–17,359 km^2^ (Fig. 1, and Table 1). However, home range size increased with the number of location days (Appendix C) and we monitored only three camels over one year with a more or less constant monitoring effort (Appendix A). These three camels used the largest ranges, all being >12,000 km^2^. The total area covered by all camels was 28,343 km^2^ or 64% of the Great Gobi A SPA area. Only 22 (0.6%) of the camel locations, all for adult female 1, fell outside of the Great Gobi A SPA, the furthest being 4.1 km from the border (Fig. 2, Appendix D).

Although individual camels showed range shifts over time, there was little indication of a generally applicable seasonal pattern (Fig. 2, Appendix E).

#### 4.1.2 Movements

Individual camels on average travelled 3.0–6.4 km/h (Appendix E). The longest distances covered within a day were 74 km within 21 h by camel 70350, 66 km within 22 h by camel 25778, 49 km within 22 h by camel 25915, and 25 km within 22 h by camel 70348.

Camels seemed sensitive to capture events. Four of five camels for which we have GPS locations within 24 h of the capture covered 64 km (camel 70350 in 9 h), 61 km (camel 25778 in 11 h), 59 km (camel 25805 in 24 h), and 46 km (camel 70348 in 17 h) following the capture event. Camel 25915, a lactating female, covered 5 km within 14 h following capture.

### 4.2 Habitat use

Our mixed models suggested preferences for intermediate values of the landscape variables. The effect was scale dependent for some predictors, while others showed the same patterns across scales (Table 2). In the different single variable models, wild camels seem to: (1) be indifferent of NDVI values and elevation at any scale when assuming a linear relationship, (2) select for intermediate NDVI values at the smallest (5 km) and largest (25 km) availability buffer, but not at intermediate scales when assuming a quadratic relationship; (3) select for intermediate elevation at all but the largest availability scales; (4) select against steep slope at all availability scales; (5) select for intermediate slope at all available scales; (6) select against distance to water within the 25 km availability buffer, but not at closer ranges; (7) select for intermediate distances to water within the 20 and 25 km availability buffer, but not at closer ranges; (8) select for *Salty Haloxylon semi*-*deserts* at all availability scales; and (9) select for *Desert shrub communities*, *Nitraria salt shrub stands*, and *Iljina deserts* at some scales but not at others.

## 5 Discussion

### 5.1 Camel range

The seven wild camels moved over a total area of 28,410 km^2^, which more or less equals the total distribution range of 21,100–33,300 km^2^ for wild camels in Mongolia estimated by various authors and based on aerial and/or ground surveys (Adiya et al., 2006, 2012; McCarthy, 2000; Reading et al., 1999; Tulgat and Schaller, 1992; Tulgat, 2002; Zhirnov and Ilyinsky, 1986). Although the collared camels did not reach as far north and south as detected in previous surveys (Adiya et al., 2012 map page 46; McCarthy, 2000 map page 107; Tulgat and Schaller, 1999 map page 15; Zhirnov and Ilyinsky, 1986 map page 60), one female camel made use of the south-eastern part of the Great Gobi A SPA that was mostly excluded from the previously mentioned wild camel distribution maps. Combining our telemetry results with the wild camel surveys of the last 10–15 years strongly suggest that wild camels still range throughout the entire Great Gobi A SPA and potentially beyond. Thus, conservation activities should extend to the entire Great Gobi A SPA, rather than focus on an assumed core area.

Movements of wild camels into China have been reported by border guards in the past, but seem to have ceased in the last decade, likely as a result of the border fence having been upgraded. Fences have previously been identified as a significant conservation concern for other far-ranging or migratory species in Mongolia, cutting them off resources in times of environmental extremes (Ito et al., 2013; Kaczensky et al., 2011a; Olson et al., 2009). Cross-border cooperation would be desirable, and ideally a trans-boundary wildlife corridor along the military zone could connect protected areas in the Mongolian and Chinese Gobi (Kaczensky et al., 2011b).

### 5.2 Mobility and disturbance potential

Movement patterns revealed that wild camels are highly mobile. Home ranges of the three most intensively monitored wild camels covered >12,000 km^2^ and had not yet reached a plateau, suggesting further increase with time. Feral dromedaries (*Camelus dromedarius*) in central Australia also ranged over extensive areas, with annual range sizes inversely correlated to average annual rainfall (Edwards et al., 2001). In Mongolia and China, wild camels have become restricted to the most unproductive areas where they show movements and range sizes similar to those of migratory or nomadic ungulates like Asiatic wild ass (Kaczensky et al. 2011a) or Mongolian gazelle (*Pocapra gutturosa*; Olson et al., 2010). However, the 74 km covered in 21 h by a wild camel came as a surprise, although similar values have been anecdotally reported for feral camels (Siebert and Newman, 1990). These long distance movements suggest that wild camels could react quickly to local food or water shortages, or avoid adverse weather conditions and other threats, but it again highlights the necessity for access to large and unfragmented habitats as shown for other migratory ungulates in Mongolia (Kaczensky et al., 2011a,b; Ito et al., 2013).

Wild camels are generally described as being extremely shy (Tulgat and Schaller, 1999; Zhirnov and Ilyinsky, 1986), having long flight distances (Reading et al., 1999), and commonly running for long distances of 35–70 km when disturbed (Indra et al., 2002; Zhirnov and Ilyinsky, 1986) and the capture related long distance movements support the anecdotal evidence. Although this behavior gives camels flexibility to react to disturbance, few areas remain, even in the Gobi, where covering 46–65 km will allow an animal to outrun human disturbance without encountering further human presence. Thus, extreme shyness and a tendency for long distance flight behavior in combination with large home ranges may well prove a limiting factor for population expansion or the recently discussed plans to re-introduce wild camels to the much smaller 9000 km^2^ Great Gobi B SPA (Adiya, 2008b). Although, Great Gobi B SPA contains large tracts of habitat comparable to Great Gobi A SPA (von Wehrden et al., 2009), its higher overall productivity results in heavier use by humans and their livestock (Kaczensky et al., 2007) and consequently a much higher disturbance potential. Given the high sensitivity to disturbance in wild camels, this factor will have to be incorporated into future habitat suitability assessments.

Livestock grazing within Great Gobi A SPA is minimal, but the number of herder families in the buffer zone has increased dramatically during the past 30 years. Furthermore, during extreme conditions such as in winter 2000–2002 or 2009–2010, the Mongolian government granted local herders grazing rights in the limited use zone of the park, particularly in the area south of the Tsagaan Bodg range (Enkhbileg et al., 2006). Our telemetry data showed that wild camels still use this area. Droughts or dzuds, also likely negatively impact wild ungulates (Kaczensky et al., 2011a) and an influx of livestock during such sensitive times may both disturb and cause direct competition with wildlife. We therefore call for alternative strategies to support local herder families during adverse weather conditions to reduce human impacts on wildlife during such catastrophic events.

Wild and domestic camels hybridize and the introgression of domestic genes into the distinct wild camel gene pool represents a major conservation concern (Enkhbileg et al., 2006; Silbermayr and Burger, 2012; Tulgat and Schaller, 1992; Zhirnov and Ilyinsky, 1986). As the number of herding families and domestic camels in the buffer zone increases, and given the far ranging nature of both domestic and wild camels, the potential for interaction and hybridization will increase. Managers and conservationists acknowledge this problem and have begun to address the issue (Enkhbileg et al., 2006). We further encourage restricting domestic camel grazing from the SPA, while implementing strong education and outreach programs that target local people.

### 5.3 Habitat requirements

Given the small number of wild camels collared and the technical problems experienced with telemetry equipment (Kaczensky et al., 2010), we can only start to understand the factors predicting wild camel habitat use. Camels seem to select habitat with intermediate values of plant productivity, elevation, and distance to water while avoiding steep slopes. However, these factors are closely coupled as plant community composition and productivity correlate with precipitation (von Wehrden and Wesche, 2007), precipitation is partly a function of elevation (high mountain ranges catch the majority of the rainfall) and relief together with geology determines the location of water points. Without a full model including predictor interactions, disentangling the importance of the individual predictors remains guesswork and we were yet unable to produce robust habitat suitability maps. Single variable analysis suggests that within Great Gobi A SPA wild camels favor areas between large depressions and high mountains, which largely confirms previous observations (Zhirnov and Ilyinsky, 1986).

Selection for intermediate values of plant productivity, expressed as selection for intermediate NDVI values and plant communities with lower productivity, came as a surprise in this extremely unproductive environment and might be explained as a trade-off between dietary and safety requirements (Creel et al., 2005; Olson et al., 2011) or quality versus quantity of available feed (Mueller et al., 2008; Singh et al., 2010). Managers and biologists have long speculated that wolf predation on camel calves represents a key factor of camel population dynamics (Indra et al., 2002; Tumennasan and Battsetseg, 2006). Concern over wolf predation even triggered wolf control in Great Gobi A SPA in the past, but with little evidence of any effect on camel recruitment (McCarthy, 2000). Unfortunately we know nothing about wild camel anti-predator behaviors or wolf habitat use in Great Gobi A SPA and can just speculate that by avoiding the most productive habitats and the vicinity of water points, camels may be able to reduce encounter rates with wolves. Thus we caution about creating additional water points (Oyunsuren and Munkhgerel, 2006) without monitoring their effect, as it may actually do little to improve camel habitat and in the worst case can result in increased predation or completion with more water dependent ungulates (Cain et al., 2012; Simpson et al., 2011).

Since human and livestock presence was restricted to the fringes of the SPA we can largely exclude avoidance of humans and livestock as a reason for selecting habitats of intermediate productivity as has been shown for Mongolian gazelles (Olson et al., 2011). Knowledge of wild camels feeding ecology is minimal, but camels seem to be able to make better use of poor feed than sheep and may go for quantity over quality. In Inner Mongolia domestic camels preferred herbaceous plants when available, but made the most extensive use of *H*. *ammodendron*, which yielded the greatest and most predictable proportion of available biomass (Mengli et al., 2006). The same may be true for wild camels in Great Gobi A SPA, where *Salty H*. *ammodendron communities* make up for 18.4% of the habitat and were selected for at all scales, despite their relative low productivity. Future research should put more emphasis on wild camel feeding ecology, ideally making use of habituated animals of the semi-captive breeding herd of wild camels (Mijiddorj, 2002b).

New telemetry technology (e.g., GlobeStar or Iridium satellite systems) have overcome past problems with the Argos satellite system (Kaczensky et al., 2010) and we have refined capture techniques to make them reasonably efficient (Walzer et al., 2012). In addition, we have compiled a comprehensive, large scale digital habitat database and developed analyses loops in R that researchers can readily apply. Thus the main limitation for more comprehensive data analysis does not lie in the analysis tools available, but rather in the small number of individuals monitored over different and limited time periods so far. We thus urge to collar additional wild camels in a systematic and coordinated manner to subsequently allow running full models to estimate resource selection function (RSF) and assess habitat suitability across the landscape so that management/conservation can be prioritized.

## Figures and Tables

**Table 1:** Wild Bactrian camels captured and monitored in the Great Gobi A SPA in Mongolia between 2002 and 2007.

Animal	Sex	Age (years)	Collar type	GPS interval (hours)	From	To	N	Days with GPS fix	100% MCP
1	Female	Adult	Argos	NA	27.10.02	27.10.03	1125	260	17,359
2	Male	Adult	GPS-Argos	Irregular	10.10.03	22.03.04	20	19	8214
25778^a^	Female	Adult	GPS-Argos	11	25.05.07	10.04.08	695	322	13,538
25805^b^	Male	Young adult	GPS-Argos	Irregular	01.06.07	11.06.07	13	11	1979
25915	Female	Young adult	GPS-Argos	11	23.05.07	06.08.08	206	131	7010
70348	Male	Adult	GPS-Argos	11	25.05.07	18.09.08	81	50	4879
70350	Male	Young adult	GPS-Argos	7	22.05.07	02.06.08	1258	378	12,740
Total					28.10.02	18.09.08	3398	1167	28,343

a Collar retrieved, animal found dead.

b Drop-off opened after 11 days.

**Table 2:** Estimates for each centered and scaled variable^a^ at five different spatial scales (availability buffers) tested individually in a binominal mixed model with animals as random factor. For the time specific NDVI value we additionally used the NDVI timeframe as a random intercept. Dark grey shading marks a significant relationship at the *P* < 0.05 level.

Variables	Availability radius around location (km)				
	5	10	15	20	25
NDVI	−0.03	−0.01	0.00	0.05	0.04
NDVI^*^2	−0.29⁎	−0.21	−0.13	−3.32	−0.24⁎
Elevation	−0.03	−0.03	−0.02	0.01	0.01
Elevation^*^2	−0.57⁎	−0.81⁎⁎	−0.73⁎⁎⁎	−0.55⁎	−0.20
Slope	−0.18⁎⁎⁎	−0.22⁎⁎⁎	−0.20⁎⁎	−0.16⁎⁎⁎	−0.13⁎⁎⁎
Slope^*^2	−0.24	−0.50⁎⁎⁎	−0.54⁎⁎	−0.65⁎⁎⁎	−0.51⁎⁎
Distance_to_water	0.00	−0.01	−0.02	−0.04	−0.06⁎
Distance_to_water^*^2	−0.03	−0.09	−0.19	−0.36⁎⁎	−0.48⁎⁎⁎

*Main habitat type*^b^					
• Higher & intermediate dry steppe/shrub communities	0.20⁎	0.16	0.15	0.21⁎	0.16
• Nitraria salt shrub stands	0.20	0.34⁎⁎	0.24⁎	0.27⁎	0.16
• Haloxylon semi-deserts	0.03	0.11	0.06	0.11	0.04
• Salty Haloxylon semi-deserts	0.17⁎	0.25⁎⁎	0.31⁎⁎⁎	0.30⁎⁎⁎	0.26⁎
• Iljinja deserts	0.27	0.48⁎⁎	0.56⁎⁎	0.63⁎⁎⁎	0.63⁎⁎⁎
• Oasis vegetation	0.47.	0.49	0.61	0.13	0.23

a Centered and scaled to a mean of zero and a standard deviation of one.

b Tested against the most common habitat type ID 12.

⁎ *P* < 0.05.

⁎⁎ *P* < 0.01.

⁎⁎⁎ *P* < 0.001.

## Glossary

DEM: digital elevation model
dzud: Mongolian expression for various situations of harsh winter conditions
glmm: generalized linear mixed model
GPS: global positioning system
IUCN: International Union for Conservation of Nature
NDVI: Normalized Difference Vegetation Index
MCP: minimum convex polygon
SPA: sprictly protected area (IUCN category I)
PQL: penalized quasi-likelihood
R: statistical software package
REML: Restricted Maximum Likelihood
SRTM: Shuttle Radar Topography Mission
WIST: Warehouse Inventory Search Tool

## References

[b0005] Adiya, Y., 2008a. Great Gobi protected area and buffer zone survey. ZSL Evolutionary Distinct & Globally Endangered (EDGE) BLOG: <http://www.edgeofexistence.org/edgeblog/?p=413>.

[b0010] Adiya, Y., 2008b. Bactrian camel update – field expedition to Gobi B. ZSL Evolutionary Distinct & Globally Endangered (EDGE) BLOG: <http://www.edgeofexistence.org/edgeblog/?p=642>.

[b0015] Adiya, Y., Dovchindorj, G., Choijin, B., 2006. Some biological and ecological aspects of the wild Bactrian camel in Mongolia. In: Adiya, Y., Lhagvasuren B., Amgalan, B. (Eds.), Proceedings of the International Workshop on Conservation and Management of the Wild Bactrian Camel 2006, 12–14 October, 2006, Ulaanbaatar, Mongolia, pp. 7–12.

[b0020] Adiya Y., Enkhbileg D., Reading R.P., Knoll E.M., Burger P. (2012). The conservation status and management of wild camels in Mongolia. Camels in Asia and North Africa. Interdisciplinary Perspectives on Their Past and Present Significance.

[b0025] Bannikov A. (1975). Wild camels in Mongolia. Oryx.

[b0030] Blumer E.S., Namshir Z., Tuya T., Mijiddorj B., Reading R.P., Mix H., Reading R.P., Enkhbileg D., Galbaatar T. (2002). Veterinary aspects of wild bactrian camel (*Camelus bactrianus ferus*) conservation in Mongolia. Ecology and Conservation of the Wild Bactrian Camel (*Camelus bactrianus ferus*). Series in Conservation Biology.

[b0035] Bolker, B., 2010. Overdispersion estimation in a binomial GLMM. [R-sig-ME] mailing list. Available at: <https://stat.ethz.ch/pipermail/r-sig-mixed-models/2011q1/005176.html>.

[b0040] Cain J.W., Owen-Smith N., Macandza V.A. (2012). The costs of drinking: comparative water dependency of sable antelope and zebra. J. Zool..

[b0045] Clark, E.L., Munkhbat, J., Dulamtseren, S., Baillie, J.E.M., Batsaikhan, N., Samiya, R., Stubbe, M., 2006. Mongolian Red List of Mammals. Regional Red List Series, vol. 1. Zoological Society of London, London (in English and Mongolian).

[b0050] Creel S., Winnie J., Maxwell B., Hamlin K., Creel M. (2005). Elk alter habitat selection as an antipredator response to wolves. Ecology.

[b0055] Development Core Team R. (2011). R: A Language and Environment for Statistical Computing.

[b0060] Dovchindorj, G., Mijiddorj, B., Adyia, Y., 2006a. The grouping of the wild camel (Camelus bactrianus ferus, Przewalskii, 1883) in Mongolia. In: Adiya, Y., Lhagvasuren, B., Amgalan, B. (Eds.), Proceedings of the International Workshop on Conservation and Management of the Wild Bactrian Camel 2006, 12–14 October, 2006, Ulaanbaatar, Mongolia, pp. 19–23.

[b0065] Dovchindorj, G., Mijiddorj, B., Adyia. Y., 2006b. Population ecology of the wild camel in Mongolia. In: Adiya, Y., Lhagvasuren B., Amgalan B. (Eds.), Proceedings of the International Workshop on Conservation and Management of the Wild Bactrian Camel 2006, 12–14 October, 2006, Ulaanbaatar, Mongolia, pp. 24–28.

[b0070] Edwards G.P., Eldridge S.R., Wurst D., Berman D.M., Garbin V. (2001). Movement patterns of female feral camels in central and northern Australia. Wildlife Res..

[b0075] Enkhbileg, D., Dovchindorj, G., Dorjgotov, A., Adiya, Y., 2006. Current situation and future management of hybrid camel (besreg) in buffer zone area of Great Gobi Protected Area “A”. In: Adiya, Y., Lhagvasuren B., Amgalan, B. (Eds.), Proceedings of the International Workshop on Conservation and Management of the Wild Bactrian Camel 2006, 12–14 October, 2006, Ulaanbaatar, Mongolia, pp. 36–46.

[b0080] Grayson R. (2007). Anatomy of the People’s gold rush in modern Mongolia. World Placer J..

[b0085] Hare J. (1997). The wild Bactrian camel *Camelus bactrianus ferus* in China: the need for urgent action. Oryx.

[b0090] Hare J. (1998). The Lost Camels of Tatary: A Quest into Forbidden China.

[b0095] Hare, J., 2008. Camelus ferus. In: IUCN 2011. IUCN Red List of Threatened Species. Version 2011.2. <www.iucnredlist.org>, (01.03.12).

[b0100] Hijmans R.J., Cameron S.E., Parra J.L., Jones P.G., Jarvis A. (2005). Very high resolution interpolated climate surfaces for global land areas. Int. J. Climatol..

[b0105] Indra P., Magash A., Batsuuri L., Reading R.P., Enkhbileg D., Galbaatar T. (2002). Problems facing wild camel conservation in Mongolia. Ecology and Conservation of the Wild Bactrian Camel (*Camelus bactrianus ferus*). Series in Conservation Biology.

[b0110] Ito T.Y., Lhagvasuren B., Tsunekawa A., Shinoda M., Takatsuki S., Buuveibaatar B., Chimeddorj B. (2013). Fragmentation of the habitat of wild ungulates by anthropogenic barriers in Mongolia. PLoS ONE.

[b0115] Kaczensky P., Enkhsaihan N., Ganbaatar O., Samjaa R., Walzer C. (2007). Identification of herder – wildlife conflicts in the Gobi B Strictly Protected Area in SW Mongolia. Explor. Biol. Resour. Mongolia (Halle/Saale, Germany).

[b0120] Kaczensky P., Ito T.Y., Walzer C. (2010). Satellite telemetry of large mammals in Mongolia: What expectations should we have for collar function?. Wildlife Biol. Practice.

[b0125] Kaczensky P., Kuehn R., Lhagvasuren B., Pietsch S., Yang W., Walzer C. (2011). Connectivity of the Asiatic wild ass population in the Mongolian Gobi. Biol. Conserv..

[b0130] Kaczensky P., Ganbataar O., Altansukh N., Enkhsaikhan N., Stauffer C., Walzer C. (2011). The danger of having all your eggs in one basket – winter crash of the re-introduced Przewalski’s horses in the Mongolian Gobi. PloS ONE.

[b0135] Kawamura K., Akiyama T., Yokota H., Tsutsumi M., Yasuda T., Watanabe O., Wang G., Wang S. (2005). Monitoring of forage conditions with MODIS imagery in the Xilingol steppe, Inner Mongolia. Int. J. Remote Sens..

[b0140] Kogan F., Stark R., Gitelson A., Jargalsaikhan L., Dugrajav C., Tsooj S. (2004). Derivation of pasture biomass in Mongolia from AVHRR-based vegetation health indices. Int. J. Remote Sens..

[b0145] McCarthy, T.M., 2000. Ecology and conservation of snow leopards, Gobi brown bears and wild Bactrian camels in Mongolia. PhD thesis, University of Massachusetts, Amherst, USA.

[b0150] Mengli Z., Willms W.D., Guodong H., Ye J. (2006). Bactrian camel foraging behaviour in a *Haloxylon ammodendron* (C.A. Mey) desert of Inner Mongolia. Appl. Anim. Behav. Sci..

[b0155] Mijiddorj B., Reading R.P., Enkhbileg D., Galbaatar T. (2002). Conservation of endangered species in sector A of Great Gobi SPA. Ecology and Conservation of the Wild Bactrian Camel (*Camelus bactrianus ferus*). Series in Conservation Biology.

[b0160] Mijiddorj B., Reading R.P., Enkhbileg D., Galbaatar T. (2002). A short introduction to the captive breeding program for wild camels in Zahuin gobi, Mongolia. Ecology and Conservation of the Wild Bactrian Camel (*Camelus bactrianus ferus*). Series in Conservation Biology.

[b0165] Mueller T., Olson K.A., Fuller T.K., Schaller G.B., Murray M.G., Leimgruber P. (2008). In search of forage: predicting dynamic habitats of Mongolian gazelles using satellite-based estimates of vegetation productivity. J. Appl. Ecol..

[b0170] Olson K.A., Mueller T., Leimgruber P., Nicolson C., Fuller T.K., Bolortsetseg S., Amanda E. Fine A.E., Lhagvasuren B., Fagan W.F. (2009). Fences impede long-distance Mongolian gazelle (*Procapra gutturosa*) movements in drought-stricken landscapes. Mongolian J. Biol. Sci..

[b0175] Olson K.A., Fuller T.K., Mueller T., Murray M.G., Nicolson C., Odonkhuu D., Bolortsetseg S., Schaller G.B. (2010). Annual movements of Mongolian gazelles: nomads in the Eastern Steppe. J. Arid Environ..

[b0180] Olson K.A., Mueller T., Kerby J.T., Bolortsetseg S., Leimgruber P., Nicolson C.R., Fuller T.K. (2011). Death by a thousand huts? Effects of household presence on density and distribution of Mongolian gazelles. Conserv. Lett..

[b0185] Oyunsuren, R., Munkhgerel D., 2006. Water supply for wild camels. In: Adiya, Y., Lhagvasuren B., Amgalan, B. (Eds.), Proceedings of the International Workshop on Conservation and Management of the Wild Bactrian Camel 2006, 12–14 October, 2006, Ulaanbaatar, Mongolia, pp. 49–54.

[b0190] Reading R.P., Mix H., Lhagvasuren B., Blumer E.S. (1999). Status of wild Bactrian camels and other large ungulates in south-western Mongolia. Oryx.

[b0195] Reading R.P., Blumer E.S., Mix H., Adiya Y. (2005). Wild bactrian camel conservation. Explor. Biol. Resour. Mongolia (Halle/Saale, Germany).

[b0200] Siebert B.D., Newman D.M.R., Richardson B.J., Walton D.W. (1990). Camelidae. Fauna of Australia. Mammalia.

[b0205] Silbermayr K., Burger P., Knoll E.M., Burger P. (2012). Hybridization: a threat to the genetic distinctiveness of the last wild old world camel species. Camels in Asia and North Africa. Interdisciplinary Perspectives on Their Past and Present Significance.

[b0210] Simpson N.O., Stewart K.M., Bleich V.C. (2011). What have we learnt about water developments for wildlife?. Calif. Fish Game.

[b0215] Singh N.J., Grachev I.A., Bekenov A.B., Milner-Gulland E.J. (2010). Tracking greenery across a latitudinal gradient in central Asia – the migration of the saiga antelope. Divers. Distrib..

[b0220] Sutherland W.J., Pullin A.S., Dolman P.M., Knight T.M. (2004). The need for evidence-based conservation. Trends Ecol. Evol..

[b0225] Tsevegmid D., Dashdorj A. (1974). Wild horses and other endangered wildlife in Mongolia. Oryx.

[b0230] Tulgat R., Reading R.P., Enkhbileg D., Galbaatar T. (2002). Causes of changes in the population size and distribution of wild bactrian camels in the 20th century. Ecology and Conservation of the Wild Bactrian Camel (*Camelus bactrianus ferus*). Series in Conservation Biology.

[b0235] Tulgat R., Schaller G.B. (1992). Status and distribution of wild Bactrian camels *Camelus bactrianus ferus*. Biol. Conserv..

[b0240] Tulgat R., Magash A., Indra R., Reading R.P., Enkhbileg D., Galbaatar T. (2002). Population biology and reproduction of wild camels (*Camelus bactrianus ferus*) in Mongolia. Ecology and Conservation of the Wild Bactrian Camel (*Camelus bactrianus ferus*). Series in Conservation Biology.

[b0245] Tumennasan, K., Battsetseg, C., 2006. Impact of grey wolves on the typical ungulates in Great Gobi Protected Area “A”. In: Adiya, Y., Lhagvasuren B., Amgalan, B. (Eds.), Proceedings of the International Workshop on Conservation and Management of the Wild Bactrian Camel 2006, 12–14 October, 2006, Ulaanbaatar, Mongolia, pp. 55–58.

[b0250] von Wehrden H., Wesche K. (2007). Relationships between climate, productivity and vegetation in southern Mongolian drylands. Basic Appl. Dryland Res..

[b0255] von Wehrden H., Wesche K., Hilbig W. (2006). Plant communities of the Mongolian Transaltay Gobi. Feddes Repertorium.

[b0260] von Wehrden H., Wesche K., Reudenbach C., Miehe G. (2006). Mapping of large-scale vegetation pattern in southern Mongolian semi-deserts – an application of LANDSAT 7 data. Erdkunde.

[b0265] von Wehrden H., Wesche K., Miehe G. (2009). Plant communities of the southern Mongolian Gobi. Phytocoenologia.

[b0270] von Wehrden H., Hanspach J., Kaczensky P., Fischer J., Wesche K. (2012). A global assessment of the non-equilibrium concept in rangelands. Ecol. Appl..

[b0275] Walzer C., Kaczensky P., Enkhbileg D., Adiya Y., Knoll E.M., Burger P. (2012). Working in a freezer: capturing and collaring wild Bactrian camels. Camels in Asia and North Africa. Interdisciplinary Perspectives on Their Past and Present Significance.

[b0280] Zevegmid D., Dawaa N. (1973). Die seltenen Großsäuger der Mongolischen Volksrepublik und ihr Schutz. Archiv für Naturschutz und Landschaftsforschung.

[b0285] Zhirnov L.V., Ilyinsky V.O. (1986). The Great Gobi National Park – A Refuge for Rare Animals in the Central Asian Deserts.

[b0290] Zhirnov L.V., Gunin P.D., Adiya Y. (2011). Wild Bactrian Camel in Central Asia. Biological Resources and Natural Conditions in Mongolia. Joint Russian–Mongolian Biological Expeditions.

