# Supplementary material for: Space and habitat use by wild Bactrian camels in the Transaltai Gobi of southern Mongolia
Source: Biol Conserv. 2014 Jan;169(100):311–8. doi: 10.1016/j.biocon.2013.11.033 (PMC3969720; doi:10.1016/j.biocon.2013.11.033)
Supplement: Supplementary data 1 [file mmc1.pdf]

APPENDIX

Appendix A: Monitoring period and number of locations by animal and month.

Year	Months	Animal						
		1	2	25778	25805	25915	70348	70350
2002	10	7						
	11	101						
	12	109						
2003	1	108						
	2	94						
	3	103						
	4	95						
	5	109						
	6	105						
	7	77						
	8	75						
	9	82						
	10	60	4					
	11		2					
	12		4					
2004	1		5					
	2		1					
	3		4					
2007	5			14		18	5	33
	6			65	13	2	1	101
	7			67		5	3	104
	8			65		1		107
	9			66		5		102
	10			68				97
	11			64		3		94
	12			68		6	5	102
2008	1			66		9	20	105
	2			63		22	6	99
	3			67		24	7	105
	4			22		20	6	99
	5					39	4	106
	6					23		4
	7					21	7	
	8					8	4	
	9						13	

Appendix B: Plant communities of the Transaltai Gobi.

Abbreviation	Plant communities: von Wehrden et al. 2009	Plant communities: von Wehrden et al. 2006	ID	Merged plant communities	% coverage	Productivity rank*
Euro.Ajan	*Allio polyrrhizi-Stipetum glareosae, eurotietosum ceratoidis*	*Eurotio ceratoidis-Zygophylletum xanthoxyli*	11	*Higher and intermediate dry steppe/shrub communities*	59.6	2
Caragana	*Oxytropidi aciphyllae-Caraganetum leucophloeae*	*Sympegmo regelii-Caraganetum leucophloeae*	12			
Cara-Anab	*Sympegmo regelii-Caragana leucophloeae*	*Sympegmo regelii-Caraganetum leucophloeae*	15			
Anabasis	*Stipo glareosae-Anabasietum brevifoliae* , typicum	*Stipo glareosae-Anabasietum brevifoliae* , typical sub-association	18			
Anab-Reau	*Stipo glareosae-Anabasietum brevifoliae* , *Reaumuria songarica* sub-association	*Stipo glareosae-Anabasietum brevifoliae, Ephedra przewalskii* sub-association	19			
Conv.	*Convolvulus gortschakovii*-community	*Convolvulus gortschakovii* community	21	*Desert shrub communities*	3.4	3
Ephe.przw	*Ephedro przewalsii-Zygophylletum xanthoxyli* associations	*Ephedro przewalski - Zygophylletum xan-thoxyli* associations	25			
Ephe.Symp	*Ephedro przewalski-Zygophylletum xan-thoxyli, sympegmetosum regelii* subassociation	NA	26			
Nitr.robo	*Ephedro przewalsii-Zygophylletum xanthoxyli, nitrarietosum roborovskii* sub-association	NA	27	*Nitraria salt shrub stands*	1.5	4
Reau.Nitr	*Salsolo passerinae-Reaumurietum soong-oricae,* *nitrarietosum sphaerocarpae* sub-association	*Reaumuria songarica-Nitraria sphaerocarpa* community	29			
Halo	*Calligono mongolici-Haloxyletum ammodendronis* , mono-dominant stands	*Calligono mongolici-Haloxyletum ammondendronis*	33	*Haloxylon semi-deserts*	10.9	4-5**
Halo.Reau	*Calligono mongolici-Haloxyletum ammodendronis, Reaumuria songorica* sub-association	*Calligono mongolici-Haloxyletum ammondendronis, Reaumuria songarica* sub-association	34	*Salty Haloxylon semi-deserts*	18.4	4-6**
Ilji	*Iljinietum regelii* association	*Iljinia regelii* community	35	*Iljinia deserts*	5.2	4-7**
Kalidium	*Nitrario sibiricae-Kalidietum gracilis, Phragmites communis* variant	*Nitrario sibiricae-Kalidietum gracilis*	36	*Oasis vegetation*	1.1	1
Popu	*Glycyrhizo uralensis-Populetum euphraticae*	*Glycyrrhizo uralensis-Populetum euphraticae*	38			
Popu-Tama	*Glycyrrhizo uralensis-Populetum euphraticae,* *Tamarix ramosissima* sub-community	NA	39			
Tama	*Tamarix ramosissima* community	*Tamarix ramosissima* community	40			
Phra.aust	*Blysmetum rufi*	*Blysmetum rufi*	41			

* 1 = highest, 7 = lowest, **habitat types are subject to a high variance in productivity

Appendix C: Size of the 100% Minimum Convex Polygon (MCP) as a function of monitoring days and date.

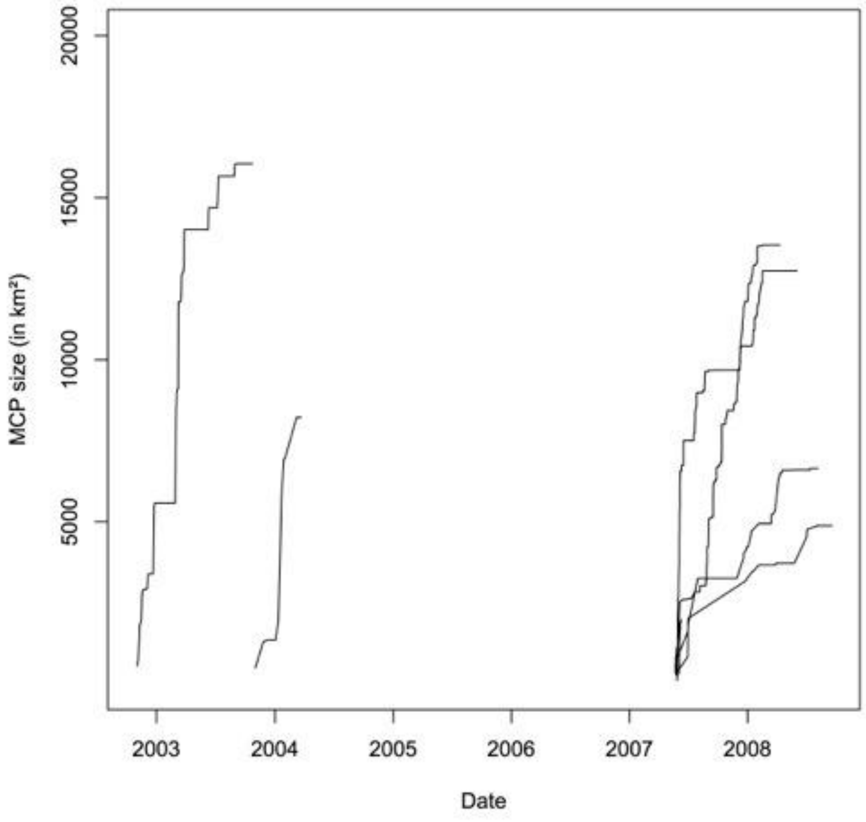

Appendix D: Locations of seven wild camels superimposed on the main habitat types in the Great Gobi A SPA.

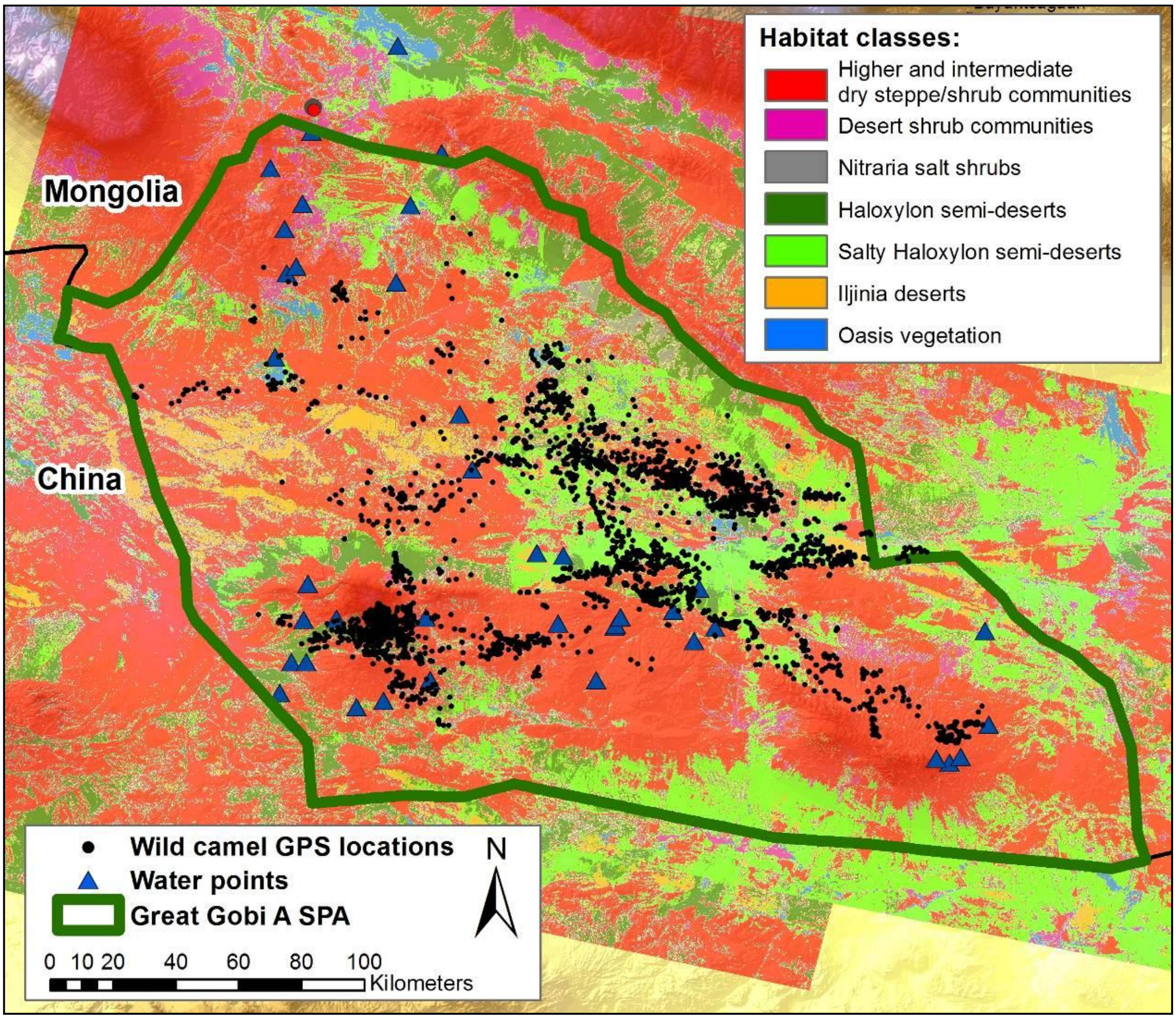

Appendix E: Net displacement to a common reference point (northern most point of SPA) of seven camels since their capture. To check for any seasonal effect, the animals collared in 2003 and 2004 were assigned to the corresponding dates in 2007 and 2008.

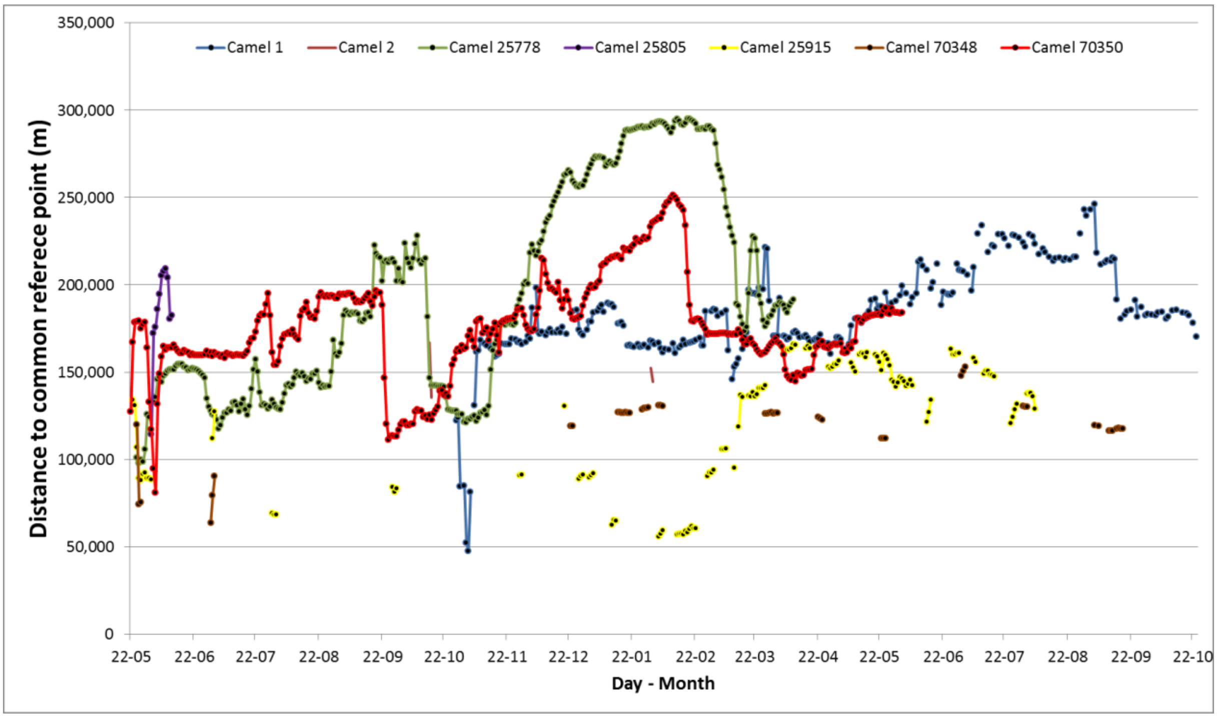

Appendix F: Daily travel distances of four wild camels monitored for

	Distances (km) travelled in a day*					
**Animal Id**	**Mean**	**Median**	**SD**	**Minimum**	**Maximum**	**N Intervalls**
25778	6.4	3.2	8.9	0.0	72.2	685
25915	5.5	3.1	8.4	0.1	53.6	82
70348	3.0	0.7	6.1	0.2	27.2	33
70350	5.7	3.2	7.7	0.0	84.7	1215

*Estimated based on distances travelled between locations spaced 21-22 hours apart and extrapolated to 24 hours by assuming a lineral relationship with time.
